# *In Vivo* Evaluation of the Visual Pathway in Streptozotocin-Induced Diabetes by Diffusion Tensor MRI and Contrast Enhanced MRI

**DOI:** 10.1371/journal.pone.0165169

**Published:** 2016-10-21

**Authors:** Swarupa Kancherla, William J. Kohler, Yolandi van der Merwe, Kevin C. Chan

**Affiliations:** 1 NeuroImaging Laboratory, University of Pittsburgh, Pittsburgh, PA, United States of America; 2 UPMC Eye Center, Ophthalmology and Visual Science Research Center, Department of Ophthalmology, School of Medicine, University of Pittsburgh, Pittsburgh, PA, United States of America; 3 Department of Bioengineering, Swanson School of Engineering, University of Pittsburgh, Pittsburgh, PA, United States of America; 4 McGowan Institute for Regenerative Medicine, University of Pittsburgh, Pittsburgh, PA, United States of America; 5 Louis J. Fox Center for Vision Restoration, University of Pittsburgh, Pittsburgh, PA, United States of America; 6 Center for the Neural Basis of Cognition, University of Pittsburgh and Carnegie Mellon University, Pittsburgh, PA, United States of America; 7 Department of Electrical and Electronic Engineering, The University of Hong Kong, Hong Kong, China; Xi'an Jiaotong University Medical College First Affiliated Hospital, CHINA

## Abstract

Visual function has been shown to deteriorate prior to the onset of retinopathy in some diabetic patients and experimental animal models. This suggests the involvement of the brain's visual system in the early stages of diabetes. In this study, we tested this hypothesis by examining the integrity of the visual pathway in a diabetic rat model using *in vivo* multi-modal magnetic resonance imaging (MRI). Ten-week-old Sprague-Dawley rats were divided into an experimental diabetic group by intraperitoneal injection of 65 mg/kg streptozotocin in 0.01 M citric acid, and a sham control group by intraperitoneal injection of citric acid only. One month later, diffusion tensor MRI (DTI) was performed to examine the white matter integrity in the brain, followed by chromium-enhanced MRI of retinal integrity and manganese-enhanced MRI of anterograde manganese transport along the visual pathway. Prior to MRI experiments, the streptozotocin-induced diabetic rats showed significantly smaller weight gain and higher blood glucose level than the control rats. DTI revealed significantly lower fractional anisotropy and higher radial diffusivity in the prechiasmatic optic nerve of the diabetic rats compared to the control rats. No apparent difference was observed in the axial diffusivity of the optic nerve, the chromium enhancement in the retina, or the manganese enhancement in the lateral geniculate nucleus and superior colliculus between groups. Our results suggest that streptozotocin-induced diabetes leads to early injury in the optic nerve when no substantial change in retinal integrity or anterograde transport along the visual pathways was observed in MRI using contrast agent enhancement. DTI may be a useful tool for detecting and monitoring early pathophysiological changes in the visual system of experimental diabetes non-invasively.

## Introduction

Diabetic retinopathy is a leading cause of acquired blindness from sequela such as edema, intraocular hemorrhage and fibrosis [1]. In 2012, the American Diabetes Association reported that 29.1 million Americans had diabetes. According to the Wisconsin Epidemiologic Study of Diabetic Retinopathy, after 20 years of diabetes mellitus, almost 99% of patients with type 1 diabetes and 60% of patients with type 2 diabetes had some degree of diabetic retinopathy. Diabetic retinopathy has caused legal blindness in 85% and 33% for the young-onset (<30 years old) and older-onset adults, respectively [2].

To date, the causes and pathophysiology of visual impairment from diabetes remain incompletely understood. Previous studies indicated that visual function may be impaired prior to physical exam findings of retinopathy in some diabetic patients [3–8]. For example, using electrophysiological testing, patients with diabetes may show altered visually evoked potentials or electroretinograms even when there is no observed retinopathy [9–11]. In addition, recent histological studies have demonstrated microstructural changes in the intracranial optic nerve of early experimental diabetes before substantial morphological alterations in the eye can be observed [12–14]. These findings suggest the early involvements of the brain’s visual system in diabetes. There is a need to investigate beyond the eye into the pathophysiological events in the brain’s visual system globally and more comprehensively, in order to better understand the exact mechanisms of visual impairments in diabetes and to guide more targeted vision preservation strategies.

In experimental diabetic animal models, streptozotocin (STZ), a glucose analog, is cytotoxic to pancreatic beta cells and can cause hyperglycemia mimicking diabetic conditions [14–18]. In this study, we hypothesize that the visual pathway is altered in the early stage of STZ–induced experimental diabetes, and such alterations can be revealed non-invasively by *in vivo* multi-modal magnetic resonance imaging (MRI). *In vivo* MRI has been increasingly employed at high magnetic field strengths to evaluate the rodent eye and brain's visual system in health and disease [19–26]. In this study, we used diffusion tensor MRI (DTI) at 7 Tesla to evaluate the white matter integrity along the visual pathway [25, 27, 28] of STZ-induced diabetic rats and sham control rats. In addition, chromium (Cr)-enhanced MRI of the retina [24, 29], and manganese (Mn)-enhanced MRI of anterograde transport [30–35] were employed to the same animals to investigate different properties of the visual system in the same disease stage. The results of this study are potentially important for determining the role of the brain’s visual pathway in early diabetes. The developed *in vivo* methods of imaging evaluation may also help with early detection for guiding more timely interventions and reducing the burden of this common disease of visual impairment [36, 37].

## Materials and Methods

### Animal Preparation

All experiments were performed in accordance with the Association of Research for Vision and Ophthalmology Statement for the Use of Animals in Ophthalmic and Vision Research. The protocol was approved by the animal care and use committee of The University of Hong Kong. Ten-week-old Sprague-Dawley female rats (N = 10) were divided into 2 groups. Five rats underwent 1 day of fasting followed by intraperitoneal injection of STZ (Sigma-Aldrich, St. Louis, MO, USA) at 65 mg/kg in 0.01M citric acid. The other 5 rats were injected with citric acid only and acted as a sham control. Body weight was measured before, and at 3, 5, 7, 14, 21 and 28 days after systemic drug administration, whereas tail-vein blood glucose level measurement and DTI of white matter integrity were performed at 1 month after systemic drug administration. All blood glucose measurements were taken in the morning before DTI acquisition without fasting. The 1-month time point was chosen in this study, as recent histological studies at 4–6 weeks after STZ injection revealed microstructural alterations in the distal optic nerve and axonal transport changes without apparent retinal ganglion cell loss in rats [12–14, 38], whereas no significant optic nerve axonal loss was found at 2 weeks after STZ injection at a similar dosage [39]. Therefore, we tested our proposed hypothesis to detect possible changes along the visual pathway early in the disease process, when retinal damages were presumed to be minimal. After DTI, hexavalent chromium, Cr(VI) (3 μL, 10 mM) was intravitreally injected into the left eye, and Cr-enhanced MRI of retinal integrity was performed 1 day later. Since more than 90% of the rat optic nerve fibers decussate at the optic chiasm to the contralateral hemisphere [40], MnCl_2_ solution (3 μL, 50 mM) was injected intravitreally into the right eye after Cr-enhanced MRI, and Mn-enhanced MRI was performed 1 day later to examine the anterograde Mn transport to the left lateral geniculate nucleus, left superior colliculus and possibly the left visual cortex.

### MRI Protocol

All MRI measurements were acquired utilizing a 7 Tesla Bruker scanner with a maximum gradient of 360 mT/m (70/16 PharmaScan, Bruker Biospin GmbH, Germany). Under inhaled isoflurane anesthesia (3% induction; 1.5% maintenance) and circulating warm water, animals were imaged using a receive-only surface coil. Scout T2-weighted images were first acquired in the coronal, transverse and sagittal planes with a rapid-acquisition-with-relaxation-enhancement (RARE) pulse sequence to position the subsequent MR images along standard anatomical orientations in a reproducible manner. For DTI, diffusion weighted images were acquired using the multi-shot spin-echo echo-planar imaging sequence, with repetition time (TR)/echo time (TE) = 3000/30 ms, field-of-view (FOV) = 32 x 32 mm^2^, matrix resolution = 128 x 128, in-plane resolution = 250 x 250 μm^2^, slice thickness = 1 mm, diffusion weighting factor (b) = 0 and 1000 s/mm^2^, diffusion time (Δ) = 15 ms, diffusion gradient duration (δ) = 5 ms, 4 shots and 30 diffusion directions. Cr-enhanced MRI and Mn-enhanced MRI were performed using 2D T1-weighted RARE sequences covering the eye and the brain with TR/TE = 475/8.8 ms, RARE factor = 4, number of averages = 26, FOV = 32 x 32 mm^2^, voxel resolution = 125 x 125 μm^2^ and slice thickness = 0.8 mm.

### Data Analysis

DTI parametric values including fractional anisotropy, axial diffusivity (**λ**_//_) and radial diffusivity (**λ**_**┴**_) were extracted from the prechiasmatic optic nerve, the optic tract and the anterior commissure of both hemispheres using DTIStudio v2.30 (Johns Hopkins University, Baltimore, MD, USA) and ImageJ v1.43u (Wayne Rasband, NIH, USA) based on the anatomical landmarks in the MR images and the rat brain atlas [41] at Bregma -2.0 mm and -3.0 mm after co-registration using AIR v5.2.5 (Roger Woods, UCLA, USA). T1-weighted signal intensities were measured in the retina bilaterally in Cr-enhanced MRI using ImageJ from the temporal ora serrata to nasal ora serrata in the slice covering the center of the eyeballs. T1-weighted signal intensities were also measured in the bilateral visual brain nuclei including the lateral geniculate nucleus, the superior colliculus and the visual cortex in Mn-enhanced MRI using ImageJ at Bregma -4.0 mm and -6.0 mm. Cr and Mn enhancements were obtained by computing the signal increase in the left retina or brain nuclei relative to the right ones. The body weight, DTI parametric values and Cr and Mn enhancements were compared between diabetic and control rats over time using repeated measures analyses of variance (ANOVA) followed by post-hoc multiple comparisons correction tests using GraphPad Prism v6.02 (GraphPad Software Inc., La Jolla, CA, USA). The blood glucose level was compared between diabetic and control rats using a two-tailed unpaired Student’s t-test. Data are represented as mean ± standard deviation. Results are considered significant when p<0.05.

## Results

**Fig 1:**
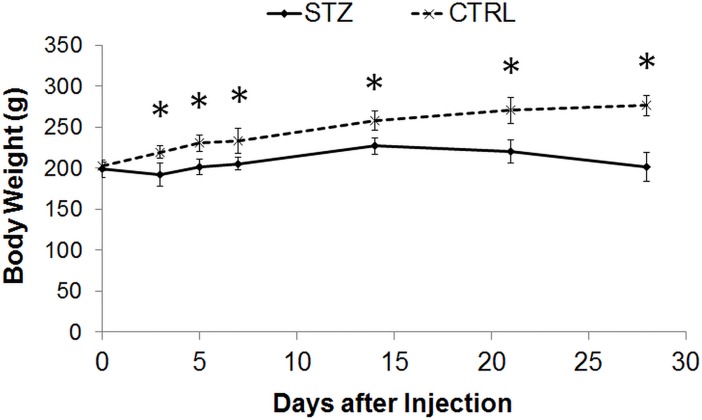
Body weights of streptozotocin-induced diabetic rats (STZ) and sham control (CTRL) rats before, and at 3, 5, 7, 14, 21 and 28 days after experimental model induction via systemic drug injection. Both groups increased their body weights over time (Repeated measures ANOVA, p<0.0001), whereas the STZ group consistently showed lower weight gain than the CTRL group after systemic drug administration (Post-hoc Sidak’s multiple comparisons correction tests, *p<0.0001).

**Fig 2:**
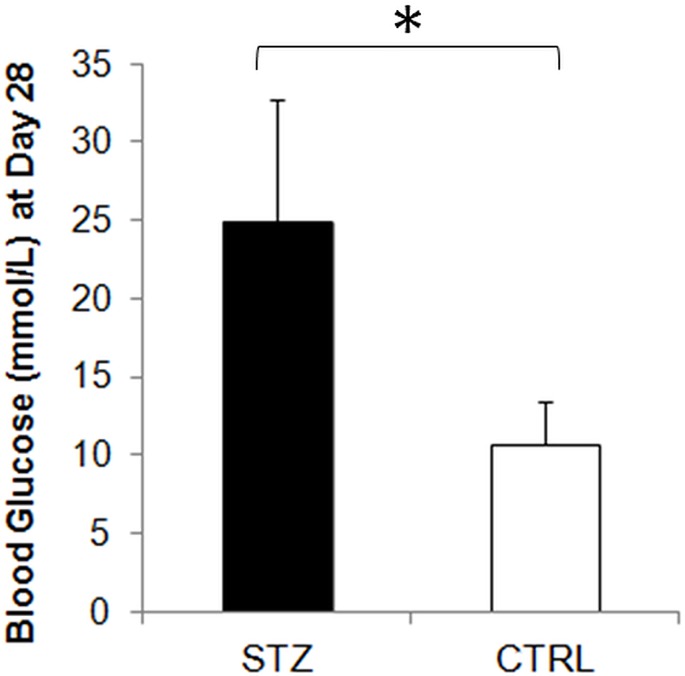
Blood glucose level at 1 month after streptozotocin (STZ) or sham control (CTRL) treatment. (Two-tailed unpaired Student’s t-test between STZ and CTRL groups, *p<0.05.)

**Fig 3:**
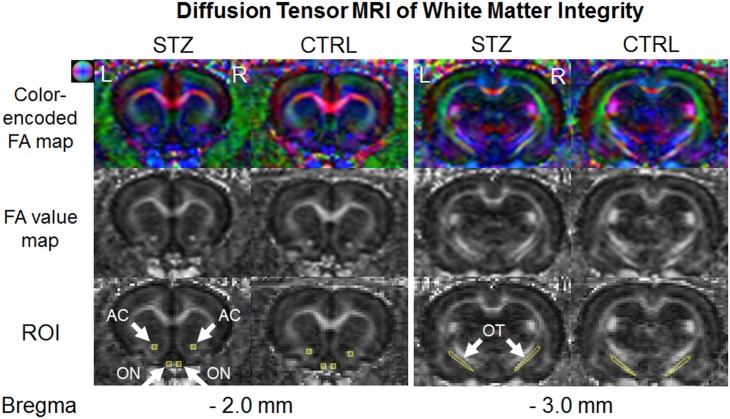
Diffusion tensor MRI of white matter integrity in the prechiasmatic optic nerve (ON), optic tract (OT) and anterior commissure (AC) (arrows). (Top row) Color-encoded fractional anisotropy (FA) directionality maps; (Middle row) FA value maps; (Bottom row) Illustrations of the regions of interest (ROI) in yellow at the corresponding Bregma locations for quantitative analyses. Note the relatively lower FA in the optic nerves of the streptozotocin (STZ) group compared to the sham control (CTRL) group. Color ball illustrates the corresponding principal diffusion directions in the color-encoded FA directionality map: blue: caudal-rostral; red: left-right; and green: dorsal-ventral.

**Fig 4:**
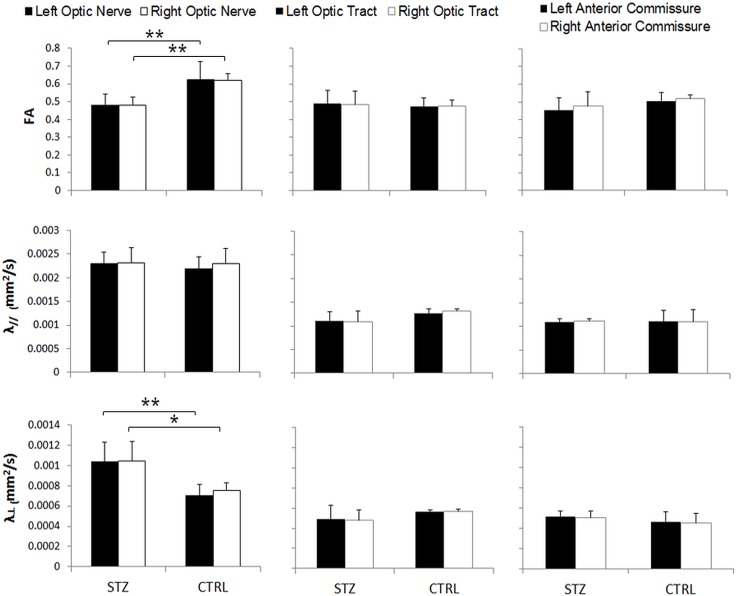
Quantitative comparisons of fractional anisotropy (FA), axial diffusivity (λ_//_) and radial diffusivity (λ_┴_) of the bilateral prechiasmatic optic nerve, optic tract and anterior commissure between streptozotocin (STZ) and sham control (CTRL) rats in diffusion tensor MRI. (Post-hoc Sidak’s multiple comparisons correction tests, *p<0.05; **p<0.01.)

**Fig 5:**
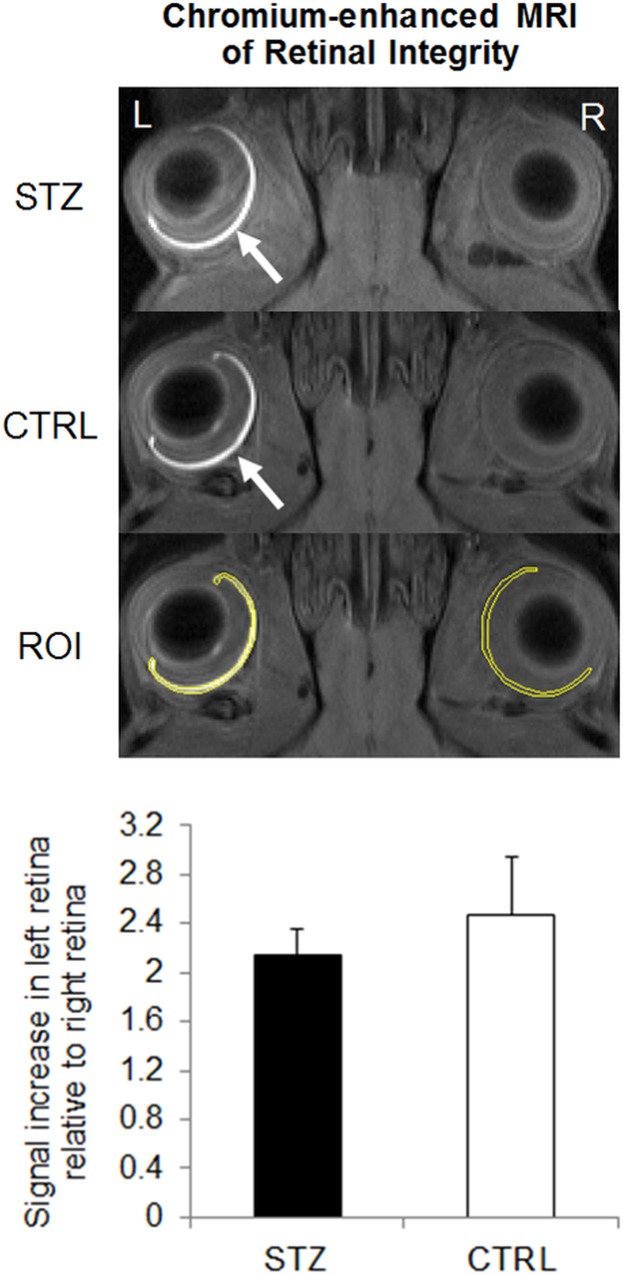
Chromium (Cr)-enhanced MRI of the retina in the streptozotocin (STZ) and sham control (CTRL) groups. (Top panel) Cr-enhanced MRI of the retina at 1 month after systemic STZ or CTRL administration, and 1 day after intravitreal Cr injection into the left eye. The regions of interest (ROI) for quantitative measurements were illustrated on both sides of the retina in yellow. Note the signal enhancements in the left retina of both groups (arrows); (Bottom panel) Quantitative comparisons of Cr signal enhancements in the retina. Significantly higher signal intensities were found in the left retina than the right retina of both STZ and CTRL groups (Post-hoc Sidak’s multiple comparisons correction tests, p<0.001). No apparent difference was found in Cr enhancement in the retina between the two groups. (Post-hoc Sidak’s multiple comparisons correction tests, p>0.05.)

**Fig 6:**
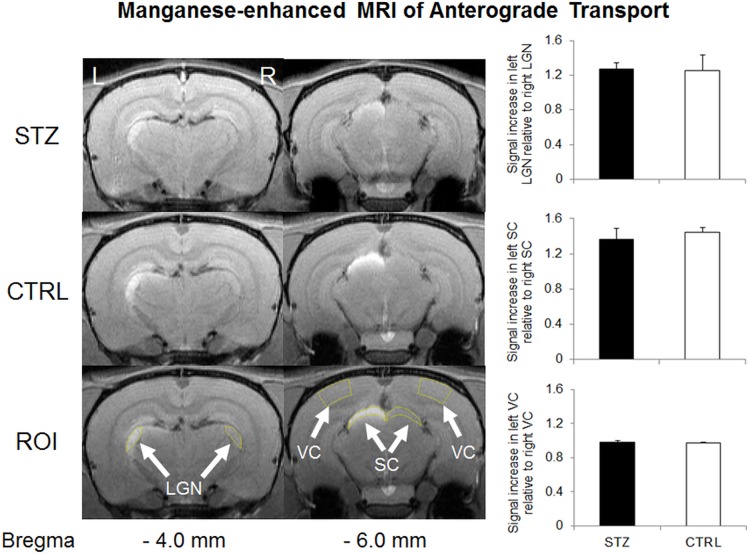
Manganese (Mn)-enhanced MRI of anterograde Mn transport along the visual pathway in streptozotocin (STZ) and sham control (CTRL) groups. (Left panel) Mn-enhanced MRI of the visual brain nuclei at 1 month after systemic STZ or CTRL administration, and 1 day after intravitreal MnCl_2_ injection into the right eye. The regions of interest (ROI) for quantitative measurements were illustrated on both sides of the lateral geniculate nucleus (LGN), superior colliculus (SC) and visual cortex (VC) in yellow (arrows). Note the signal enhancements in the left lateral geniculate nucleus and left superior colliculus of both groups; (Right panel) Quantitative comparisons of Mn signal enhancements in the visual brain nuclei. Significantly higher signal intensities were found in the left LGN and SC than the right ones in both STZ and CTRL groups (Post-hoc Sidak’s multiple comparisons correction tests, p<0.01). No apparent difference in signal intensity was found between left and right VC in either group (Post-hoc Sidak’s multiple comparisons correction tests, p>0.05). No apparent difference in Mn enhancement was found in the lateral geniculate nucleus, the superior colliculus or the visual cortex between the two groups (Post-hoc Sidak’s multiple comparisons correction tests, p>0.05).

STZ-induced diabetic rats showed a significantly smaller weight gain than sham control rats throughout the 1-month experimental period (Fig 1). The blood glucose level was also significantly higher in the diabetic rats than the control rats prior to MRI experiments at 1 month after systemic drug administration (Fig 2). DTI revealed significantly lower fractional anisotropy and significantly higher radial diffusivity in the prechiasmatic optic nerves of the diabetic rats compared to control rats at 1 month after experimental model induction (Figs 3 and 4). No apparent difference in axial diffusivity was observed in the optic nerve between groups (Fig 4). No apparent DTI difference was either observed in the optic tract or the anterior commissure between groups (Fig 4). Intravitreal Cr injection to the left eye enhanced the left retina of both diabetic and control groups at 1 day after Cr administration and 1 month after experimental model induction (Fig 5). No apparent difference was found in Cr enhancement in the retina between diabetic and control groups. Intravitreal Mn injection to the right eye enhanced the subcortical visual nuclei in the left hemisphere at 1 day after Mn administration and 1 month after experimental model induction to both groups (Fig 6). No apparent difference in Mn enhancement was found in the lateral geniculate nucleus, the superior colliculus or the visual cortex between diabetic and control groups. No apparent difference was observed in the cross-sectional diameter of the prechiasmatic optic nerve between experimental and control groups using scout T2-weighted MRI at 125μm x 125μm in-plane resolution (data not shown).

## Discussion

The STZ-induced diabetic rat model has been shown to cause abnormal visual function in the brain similar to diabetic patients [42]. While this model may also lead to significant retinal thinning and inner retinal dysfunction few months after experimental induction [16–18, 43], recent histological studies have demonstrated significant axonal loss, myelin alterations, and astrocyte reactivity increase only in the intracranial but not intraorbital portion of the optic nerve after 6 weeks of STZ-induced experimental diabetes, with no substantial morphological alterations in the retina or superior colliculus [12–14]. In this study, our experimental diabetic rat model showed a smaller weight gain and a higher blood glucose level in concurrence with the literature [44–47]. In addition, our diabetic rats showed significant DTI changes in the prechiasmatic optic nerve compared to the sham control rats at 4 weeks after STZ injection, with no statistically significant changes in the retina or visual brain nuclei by contrast-enhanced MRI. No apparent DTI difference was either observed in the intraorbital portion of the optic nerve (p>0.05) (data not shown). These initial results coincided with recent histological observations which suggest that axoglial alterations at the distal portion of the optic nerve could be among the first structural changes in the diabetic visual pathway. Apart from the direct damaging effects from hyperglycemia, it is also possible that the observed DTI changes were partly resulted from diabetes-related physiological changes such as perturbations of cerebral blood flow and vascular permeability in the optic nerve [48–50]. No apparent difference was observed in the optic tract or the anterior commissure in concurrence with a similar DTI study using voxel-based analysis to STZ-induced male rats [51]. DTI may offer sensitive and important *in vivo* imaging biomarkers to detect compromise of the integrity in the brain’s visual pathway in early experimental diabetes.

Mn-enhanced MRI has been used to detect impaired intraretinal Mn uptake [21, 22] and reduced axonal transport in other sensory systems such as the olfactory pathway [52] in STZ-induced diabetic rodents. Although previous histological studies also demonstrated axonal transport impairments between the retina and the subcortical visual nuclei at 6 weeks and beyond after systemic STZ administration in rats [12–14, 53], our present *in vivo* Mn-enhanced MRI settings did not detect such anterograde Mn transport alteration along the retinogeniculate and retinocollicular pathways at 4 weeks after STZ injection. This may be explained by the small effect size of anterograde transport deficits, if present, in early experimental diabetes. Previous Mn-enhanced MRI and DTI experiments indicated that the anterograde Mn transport is relatively more sensitive to axial diffusivity changes than radial diffusivity changes in the injured visual pathway [25]. In addition, among the DTI parameters, the fractional anisotropy has been suggested to reflect the overall microstructural integrity of the white matter, whereas axial diffusivity and radial diffusivity changes may be more sensitive to axonal and myelin injuries, respectively [54–56]. In this study, our Mn-enhanced MRI and DTI results appeared to corroborate one another, with neither Mn enhancement nor axial diffusivity showing statistically significant differences between diabetic and control groups along the visual pathway. Instead, the lower fractional anisotropy in the optic nerve appeared to be mainly attributed to the higher radial diffusivity in the diabetic rats, suggestive of pathophysiological changes that affect radial diffusivity (e.g. gliosis, myelin damage and edema) in the brain’s visual pathway during early experimental diabetes at 4 weeks after STZ administration [50]. More detailed studies are envisioned to delineate the exact microstructural contributions to diabetes in the optic pathway by combining advanced *in vivo* diffusion MRI measures [57–59] with visual outcomes and histological correlations across time and in larger cohorts. We may also consider examining the potential involvements of trans-neuronal degeneration in the visual cortex by diffusion kurtosis MRI, given this extended imaging technique can represent water diffusion properties more precisely than DTI alone, and is potentially more sensitive to microstructural complexities particularly in the gray matter [60–62].

The retina has the highest level of long-chain polyunsaturated fatty acids in the body [63, 64], and the lipid contents may be altered in the retina of STZ-induced diabetic rats [65, 66]. Although Cr(VI) may enhance oxidizable lipids in the retina and cerebral white matter in T1-weighted MRI [29, 67], our Cr-enhanced MRI experiment did not detect substantial difference in retinal enhancement between STZ-induced diabetic rats and control rats at 4 weeks after experimental model induction. However, it remains inconclusive to directly infer our current DTI and Cr-enhanced MRI results to primary optic nerve damages in diabetes, given the initial evidence on early and/or transient changes in the retina of some STZ-induced diabetic models [46, 47, 68–70]. It is also possible that the current Cr-enhanced MRI settings may not be sensitive enough to detect specific retinal changes in early experimental diabetes. Whether the optic nerve damages were, at least in part, secondary to the early retinal injuries warrants further investigations via longitudinal studies and further optimization of ocular imaging techniques for early disease detection [19–23].

This study has several limitations, the first being the use of only female rats for examining diabetes-related changes in the visual system. Rodents may exhibit gender differences in STZ sensitivity [44, 45, 71, 72], and a recent DTI study in STZ-induced male rats only showed a diffuse pattern of reduced fractional anisotropy unilaterally near the prechiasmatic optic nerve using voxel-based analysis at a similar time point [51]. Future studies should evaluate the gender effects in the diabetic brains by examining both males and females in different experimental diabetic models. On the other hand, our current *in vivo* MRI examinations were limited to one early experimental time point rather than across time, as an initial step to test their feasibility and sensitivity of early detection using a minimal amount of animals, and to avoid confounds from multiple intraocular injection injuries, residual signal enhancements and accumulated toxicity from multiple Cr or Mn injections. Future studies may elucidate when different properties of diabetic changes may begin to occur in the eye and the brain's visual system using separate groups of experimental animals with varying disease severity, and via longitudinal MRI assessments using the less toxic Mn or Cr chelates [73] or other non-invasive measurements. Another avenue of investigation may include more specific serum biochemical indices in addition to the blood glucose level to determine the metabolic states of diabetes in our experimental model more comprehensively [74]. Although a previous study using water maze paradigm showed cognitive impairment in the diabetic rats at 12 weeks after STZ injection at similar doses [75], we did not measure the visual function or behavior in our Sprague-Dawley rats during early experimental diabetes mainly because of the intrinsically low visual acuity in albino rats [76]. Future studies may employ multi-modal MRI and visuomotor behavioral assessments [76, 77] using pigmented rodents, to evaluate how alterations in structural and physiological MRI characteristics in the diabetic eye and brain may reflect psychophysical changes under different STZ dosages mimicking different pathological conditions and severity in human diabetes.

Non-invasive DTI has been used to detect white matter deficits and their relationships with neurocognitive tests in diabetic patients [78, 79]. In particular, patients with long-standing type 1 diabetes exhibited reduced fractional anisotropy in the posterior corona radiata and optic radiation, which correlated with the duration of diabetes [78]. Given the current experimental DTI findings, future human DTI studies are envisioned that examine the visual pathway integrity and their relationships with clinical ophthalmic imaging evaluation, ocular MRI assessments [80] and visual function outcomes in the early stages of diabetic patients, in order to probe the early pathophysiological events in the visual system and the corresponding eye-brain-behavior relationships in diabetes.

## Conclusion

Our results suggest that STZ-induced diabetes leads to early white matter injury in the prechiasmatic optic nerve observable by DTI, when no substantial change was detected in the retina, superior colliculus, lateral geniculate nucleus or visual cortex by Cr-enhanced MRI or Mn-enhanced MRI at 7 Tesla. These results may provide an initial step to determine the early pathophysiological events of diabetes in the brain’s visual pathway, and guide strategies for more targeted intervention to prevent further vision loss from diabetes.
